# Supplementary material for: Comparative study of neighboring Holm oak and olive trees-belowground microbial communities subjected to different soil management
Source: PLoS One. 2020 Aug 11;15(8):e0236796. doi: 10.1371/journal.pone.0236796 (PMC7418964; doi:10.1371/journal.pone.0236796)
Supplement: S1 File — (PDF) [file pone.0236796.s001.pdf]

Comparative study of neighboring Holm oak and olive trees-belowground microbial communities subjected to different soil management

Antonio J. Fernández-González [1], Nuria M. Wentzien [1], Pablo J. Villadas [1], Ana V. Lasa [1], Carmen Gómez-Lama Cabanás [2], Jesús Mercado-Blanco [2] and Manuel Fernández-López [1,*]

Supporting information

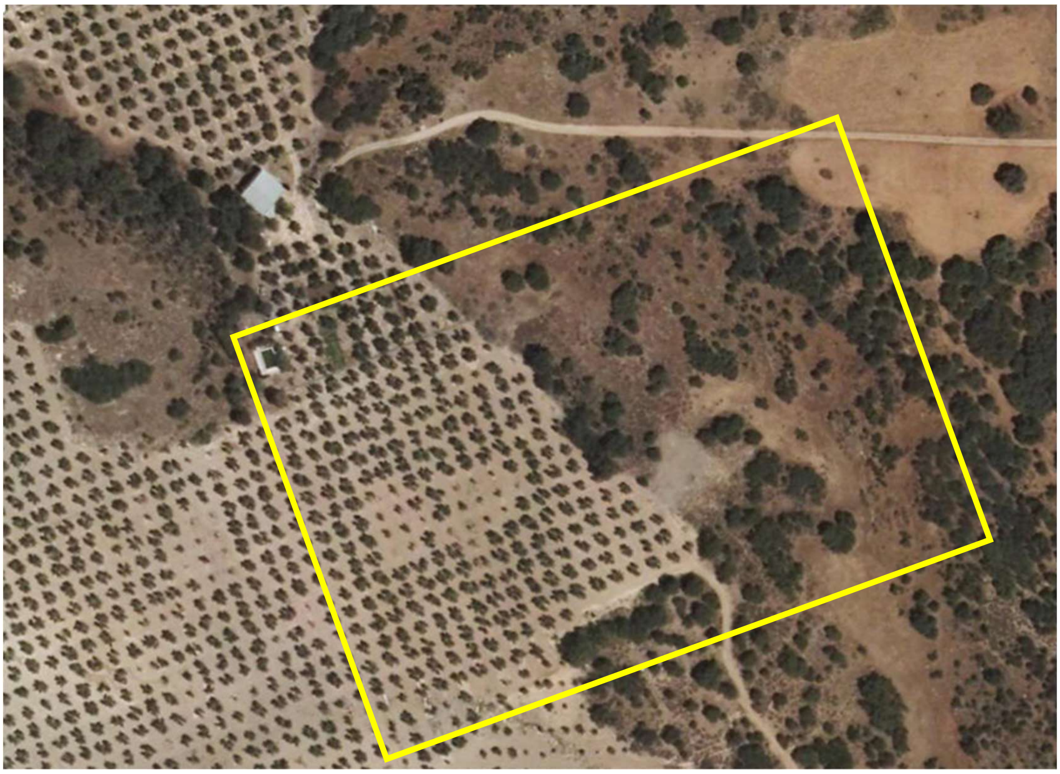

GPS coordinates: 37° 53' 16.8" N, 3° 38' 06.1" W; South-East Spain. Satellite image obtained from https://earthexplorer.usgs.gov/

**Parameter**	OLB1	OLB2	OLB4	OLB5	OLB6	HOB1	HOB2	HOB4	HOB5	HOB6
CEC (meq /100 g)	25.7	24.6	22.6	23.0	25.5	33.3	31.4	28.5	39.3	39.3
Ca (mEq /100 g)	20.7	19.4	17.3	16.8	20.8	28.1	27.1	23.5	32.4	34.1
Mg (mEq /100 g)	3.9	3.7	3.7	4.6	3.5	4.1	3.1	3.9	5.3	3.8
Na (mEq /100 g)	0.41	0.41	0.39	0.46	0.42	0.4	0.44	0.38	0.42	0.38
K (mEq /100 g)	0.71	1.04	1.17	1.08	0.75	0.78	0.69	0.78	1.17	0.99
Carbonates (%)	40.8	41.8	44.6	41.3	44.4	17.7	10.2	27.1	4.2	3.8
Active lime (%)	12.9	13.1	13.1	4.5	13.4	3.5	2.6	5.6	0.7	0.4
Phosphorus (mg/Kg)	4.5	6.6	18.1	10.1	7.3	5.7	4.3	4.0	3.9	4.2
Organic matter (%)	1.4	1.5	2.1	1.6	1.6	7.6	5.5	4.4	8.2	8.4
Organic nitrogen (%)	0.08	0.09	0.14	0.11	0.10	0.42	0.32	0.24	0.46	0.48
pH 1 / 2.5	8.5	8.4	8.4	8.6	8.5	8.3	8.3	8.4	8.2	8.3
pH (in KCl)	7.4	7.5	7.5	7.5	7.5	7.4	7.4	7.4	7.2	7.2
Exch. K (mg/Kg)	264	376	415	410	288	268	235	293	455	358
Clay (%)	34.8	29.1	28.6	26.0	39.5	15.1	12.5	15.6	19.1	16.0
Sand (%)	17.1	17.7	22.8	22.3	14.5	34.8	37.0	29.5	20.9	21.7
Silt (%)	48.1	53.2	48.6	51.7	46.0	50.1	50.5	54.9	60.0	62.3

Determination of soil parameters.

Texture of soil samples was measured by Bouyoucos hydrometer method (Bouyoucos, 1962). Potentiometric method (Willard *et al.*, 1974; Bates, 1983) was used for the determination of pH. Available water measurement was carried out by gravimetry after drying at a maximum temperature of 105 °C (Gardner, 1986). The method of electrical conductivity was used for determine the salinity. Quantification of total organic carbon was made by volumetric techniques with wet oxidation at controlled temperature (Mebius, 1960). Total nitrogen determination was performed with the Kjeldahl method (Bremner, 1965). Soluble P was measured by Bray method (Bray and Kurtz, 1945), and quantification is performed by colorimetry. Determination exchangeable bases soil was made by spectrometry using ammonium acetate.

Bates, RG. Determination of pH, Wiley, New York. (1983).

Bouyoucos, GS. Hydrometer method improved for making particle size analysis of soils. *Agron. J.* **54**, 464-465 (1962).

Bray, RH and Kurtz, LT. Determination of total, organic and available form of phosphorus in soil. *Soil Sci*. **59**, 360-361 (1945).

Bremner, JM. Inorganic forms of nitrogen. p. 1179-1237. *In* C.A. Black et al. (ed.) Methods of soil analysis. Part 2. Agron. Monogr. 9. ASA, Madison, WI (1965).

Gardner WH. Water content. In Methods of Soil Analysis, Part 1, Klute ed. Am. Soc. Agronomy, Soil Sci. Soc. Am., pp. 493-544 (1986).

Mebius LJ. A rapid method for the determination of organic carbon in soil. *Anal. Chem. Acta* **22**, 120-124 (1960).

Willard HH, Merrit LL, Dean JA. Instrumental methods of analysis. 5th edition Van Nostrand. (1974).

S1 Fig. Location and characteristics of the sampled soils. OLB: Bulk soil of the Olive orchard; HOB: Bulk soil of the holm-oak site; CEC: cation exchange capacity; Phosphorus determined is the assimilable phosphorus; Exch.K: exchangeable potassium. Yellow square indicates the sampling area.

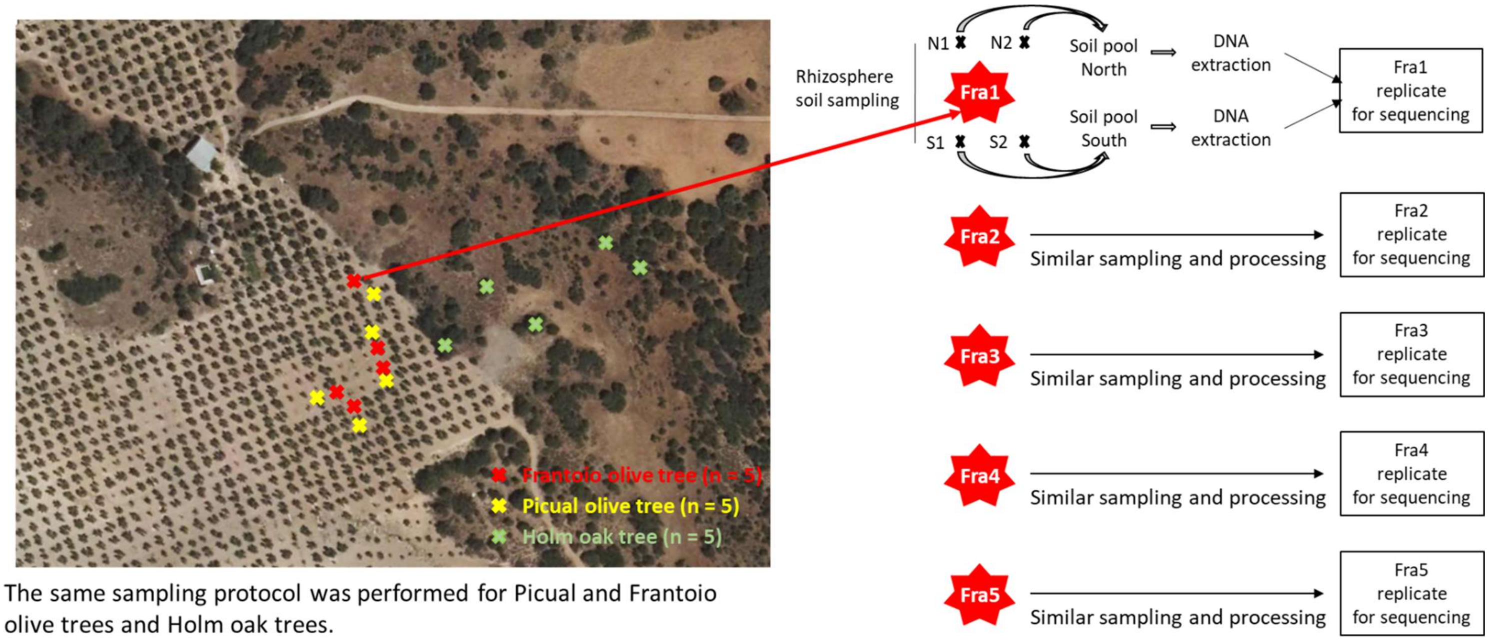

S2 Fig. Scheme of the sampling methodology followed. Fra1 corresponds to the first tree (replicate) from 'Frantoio' olive cultivar. Satellite image obtained from https://earthexplorer.usgs.gov/

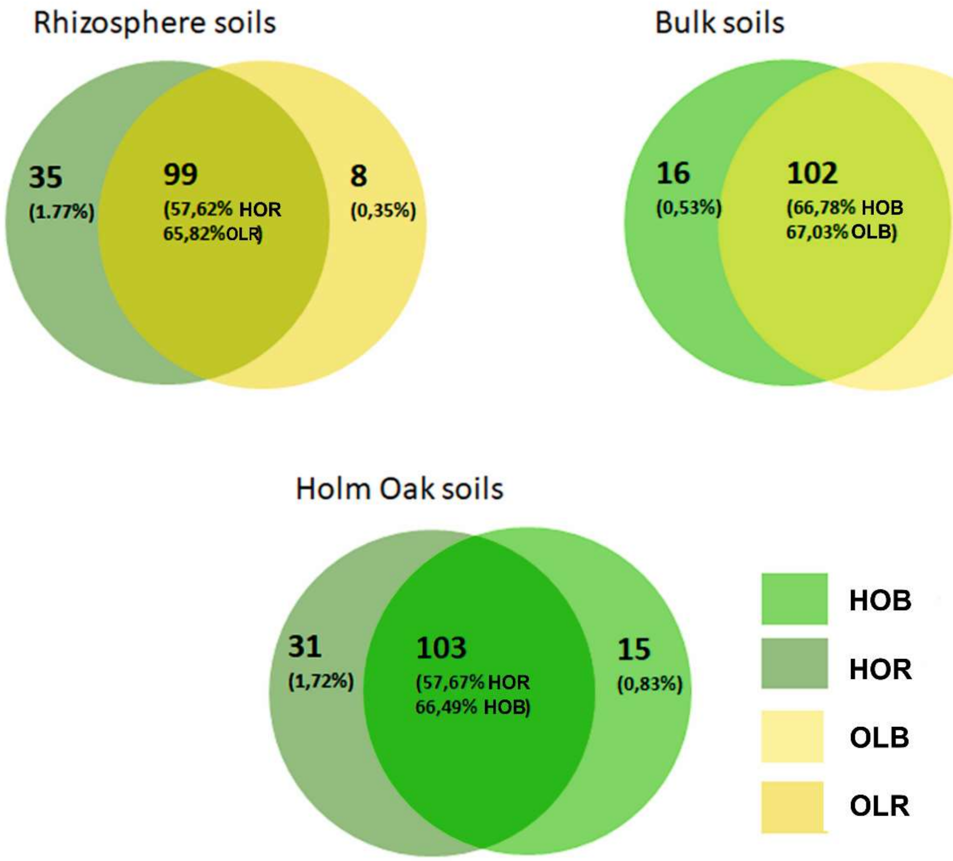

S3 Fig. Venn diagrams showing the specific and shared core bacteriome genera among the statistically different treatments. HOB: Holm-oak bulk soil, HOR: Holm-oak rhizosphere, OLB: olive bulk soil, OLR: Frantoio and Picual rhizospheres as a unique treatment (Olive rhizosphere). Bold numbers are the number of OTUs and percentages between brackets indicate the relative abundance of these OTUs in each set.

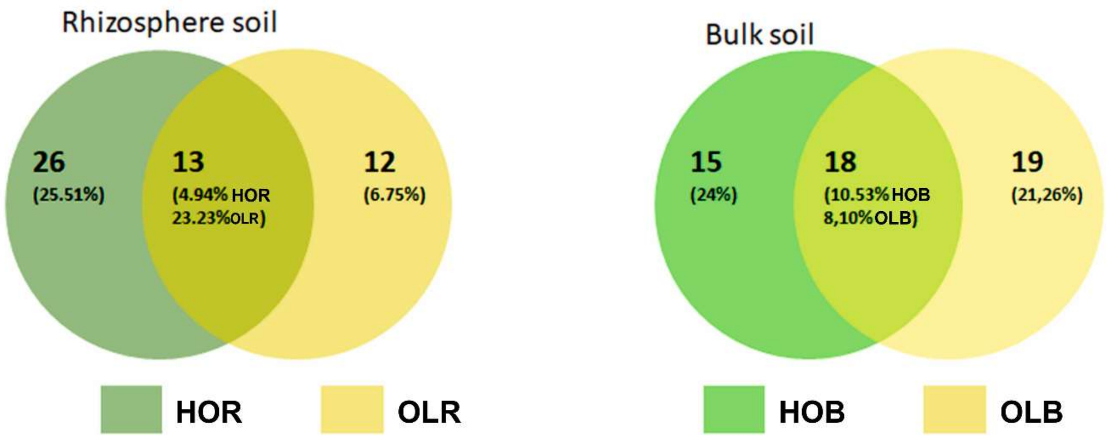

S4 Fig. Venn diagrams showing the specific and shared core mycobiome genera among the statistically different treatments. HOB: Holm-oak bulk soil, HOR: Holm-oak rhizosphere, OLB: olive bulk soil, OLR: Frantoio and Picual rhizospheres as a unique treatment (Olive rhizosphere).

S1 Table. Number of raw and overlapped reads from sequencing, number and percentage of final sequences after all the trimming process, as well as number of OTUs of each replicate. HOB: Holm-oak bulk soil; OLB: olive bulk soil; HOR: Holm-oak rhizosphere; Fra: Frantoio rhizosphere; Pic: Picual rhizosphere.

Bacteria

Samples	Raw reads	Overlapped reads	Final sequences	% final	OTUs
HOB1	58480	52863	36958	63.2%	1544
HOB2	54233	48577	34446	63.5%	1411
HOB4	45117	39975	27924	61.9%	1450
HOB5	54793	49592	36395	66.4%	1569
HOB6	54591	49185	34280	62.8%	1490
OLB1	46389	41465	32839	70.8%	1362
OLB2	46406	41290	32050	69.1%	1534
OLB4	43618	39986	31144	71.4%	1423
OLB5	48813	44451	34566	70.8%	1490
OLB6	48338	43642	33911	70.2%	1457
HOR1	23233	21011	14621	62.9%	1293
HOR2	59815	53932	37507	62.7%	1274
HOR4	56019	49758	36825	65.7%	1398
HOR5	57778	51542	35559	61.5%	1441
HOR6	53700	48955	33080	61.6%	1382
Fra1	39074	34919	27497	70.4%	1320
Fra2	43079	38227	29622	68.8%	1456
Fra4	43445	39778	29801	68.6%	1472
Fra5	57597	52214	40278	69.9%	1562
Fra6	39280	35670	28472	72.5%	1416
Pic1	41536	37531	29930	72.1%	1454
Pic2	44133	39318	31914	72.3%	1431
Pic4	38689	34371	28229	73.0%	1338
Pic5	55771	50730	40762	73.1%	1491
Pic6	44226	39939	32013	72.4%	1417
Total	1198153	1078921	810623	67.7%	

Fungi

Samples	Raw reads	Overlapped reads	Final sequences	% final	OTUs
HOB1	36406	31754	26283	72.19%	376
HOB2	38520	35082	30863	80.12%	284
HOB4	25786	23301	21000	81.44%	163
HOB5	40838	35952	31149	76.27%	371
HOB6	41715	36872	30676	73.54%	336
OLB1	40615	34513	30849	75.95%	183
OLB2	36207	30289	27071	74.77%	327
OLB4	59867	49388	43605	72.84%	351
OLB5	53000	44555	39113	73.80%	346
OLB6	35712	29207	25978	72.74%	275
HOR1	39543	35364	31684	80.13%	246
HOR2	52014	46872	42539	81.78%	201
HOR4	45259	40403	36907	81.55%	190
HOR5	47970	42499	37968	79.15%	260
HOR6	50228	44939	40951	81.53%	207
Fra1	36988	31713	27842	75.27%	216
Fra2	45545	39124	33772	74.15%	264
Fra4	50807	44058	39752	78.24%	280
Fra5	34482	29023	26133	75.79%	268
Fra6	30763	28120	25360	82.44%	210
Pic1	35665	28953	25733	72.15%	267
Pic2	39441	34898	28311	71.78%	265
Pic4	42714	37177	32907	77.04%	235
Pic5	45898	37821	33335	72.63%	312
Pic6	35329	29296	25866	73.21%	239
Total	1041312	901173	795647	76.41%	

S2 Table. Bacterial genera showing strong correlation with the significant physicochemical parameters. Genera showing differences in any of the comparisons are in bold type.

Genus	statistic	p-value	*rho*	Parameter
Actinoplanes	878.70	3.12E-04	0.66	OM
Arthrobacter	438.24	2.62E-07	0.83	pH
Asanoa	698.84	3.29E-05	0.73	OM
	4260.57	5.90E-04	-0.64	pH
Bradyrhizobium	842.53	2.08E-04	0.68	OM
candidate_division_WPS-1	**834.76**	**1.90E-04**	**0.68**	**pH**
Chelativorans	671.71	2.21E-05	0.74	OM
Chelatococcus	419.68	1.65E-07	0.84	OM
	4374.07	1.72E-04	-0.68	pH
Chthonomonas/Armatimonadetes_gp3	934.40	5.61E-04	0.64	pH
	4906.11	3.52E-09	-0.89	OM
Geminicoccus	1032.45	1.42E-03	0.60	OM
Gemmatimonas	**761.29**	**7.72E-05**	**0.71**	**pH**
	**4785.53**	**1.45E-07**	**-0.84**	**OM**
Glycomyces	4283.23	4.68E-04	-0.65	OM
Gp25	4595.27	7.59E-06	-0.77	OM
Gp3	473.75	5.98E-07	0.82	OM
Gp6	**4406.71**	**1.16E-04**	**-0.69**	**OM**
Labrys	601.37	7.16E-06	0.77	OM
Litorilinea	882.72	3.26E-04	0.66	OM
Microvirga	874.69	2.99E-04	0.66	OM
Mycobacterium	**597.35**	**6.69E-06**	**0.77**	**OM**
	**4605.77**	**6.34E-06**	**-0.77**	**pH**
Nitrospira	4278.09	4.94E-04	-0.65	OM
Planctopirus	922.15	4.95E-04	0.65	OM
	4238.53	7.34E-04	-0.63	pH

S3 Table. Fungal genera showing strong correlation with the significant physicochemical parameters. OM: organic matter; Exch.K: exchangeable potassium. The genera showing differences in any of the comparisons are in bold type.

Genus	statistic	p-value	rho	Parameter
Acrophialophora	835.88	1.93E-04	0.68	OM
	4319.85	3.17E-04	-0.66	pH
Alternaria	**897.16**	**3.81E-04**	**0.65**	**pH**
Arthrobotrys	4542.65	1.78E-05	-0.75	OM
Ascorhizoctonia	981.16	8.88E-04	0.62	pH
Bradymyces	980.08	8.79E-04	0.62	pH
	4274.11	5.14E-04	-0.64	OM
Budhanggurabania	1016.90	1.24E-03	0.61	pH
Coniocessia	4491.40	3.78E-05	-0.73	pH
	693.78	3.06E-05	0.73	OM
Coniosporium	**946.15**	**6.31E-04**	**0.64**	**pH**
Coprinus	878.65	3.12E-04	0.66	Exch.K
Dominikia	4683.31	1.49E-06	-0.80	OM
Entoloma	4419.75	9.84E-05	-0.70	OM
Exophiala	997.11	1.03E-03	0.62	pH
Fusarium	**692.86**	**3.02E-05**	**0.73**	**pH**
	**4498.15**	**3.44E-05**	**-0.73**	**OM**
Geminibasidium	4321.76	3.11E-04	-0.66	pH
Geosmithia	4197.34	1.09E-03	-0.61	Exch.K
Glomus	**4532.31**	**2.08E-05**	**-0.74**	**OM**
Halosarpheia	4233.81	7.69E-04	-0.63	OM
Hyaloseta	834.77	1.90E-04	0.68	pH
Ilyonectria	**4282.11**	**4.74E-04**	**-0.65**	**OM**
Lempholemma	1016.17	1.23E-03	0.61	pH
Lophiostoma	1021.72	1.29E-03	0.61	OM
Lophiotrema	965.63	7.65E-04	0.63	pH
	4367.40	1.86E-04	-0.68	OM
Lotinia	796.06	1.20E-04	0.69	pH
	4417.11	1.02E-04	-0.70	OM
Malassezia	4241.91	7.10E-04	-0.63	OM
Malbranchea	4568.49	1.18E-05	-0.76	OM
Metacordyceps	4337.54	2.61E-04	-0.67	OM
Peziza	651.81	1.63E-05	0.75	OM
	4190.01	1.16E-03	-0.61	pH
Podospora	945.58	6.28E-04	0.64	OM
Pyrenochaeta	4207.05	9.92E-04	-0.62	OM

Rhinocladiella	1014.51	1.21E-03	0.61	OM
	4416.37	1.03E-04	-0.70	pH
Schwanniomyces	1004.43	1.10E-03	0.61	Exch.K
Setophaeosphaeria	892.78	3.64E-04	0.66	pH
Solicoccozyma	**4657.31**	**2.48E-06**	**-0.79**	**OM**
Stachybotrys	**974.54**	**8.33E-04**	**0.63**	**pH**
	**4645.78**	**3.09E-06**	**-0.79**	**OM**
Subramaniula	**825.36**	**1.71E-04**	**0.68**	**pH**
	**4586.96**	**8.73E-06**	**-0.76**	**OM**
Talaromyces	953.83	6.81E-04	0.63	OM
Thanatephorus	967.48	7.78E-04	0.63	pH
	4324.62	3.01E-04	-0.66	OM
Trichophaea	625.77	1.08E-05	0.76	OM
Tuber	**932.95**	**5.53E-04**	**0.64**	**OM**
Wallemia	4323.61	3.05E-04	-0.66	OM
Xanthomendoza	884.71	3.34E-04	0.66	Exch.K
